# Frequency of lipoprotein(a) testing and its levels in Pakistani population

**DOI:** 10.21542/gcsp.2024.37

**Published:** 2024-08-01

**Authors:** Hijab Batool, Madeeha Khan, Quratul Ain, Omar R. Chughtai, Muhammad D. Khan, Mohammad I. Khan, Fouzia Sadiq

**Affiliations:** 1 Chemical Pathology, Chughtai Institute of Pathology, Lahore, Pakistan; 2 Directorate of Research, Shifa Tameer-e-Millat University, Pitras Bukhari Road, H-8/4, Islamabad 44000, Pakistan; 3 Atta ur Rehman School of Applied Biosciences, National University of Sciences and Technology, H-12, Islamabad 44000, Pakistan; 4 Translational Genomics Laboratory, Department of Biosciences, COMSATS University Islamabad, Islamabad, Pakistan; 5 Shifa Tameer-e-Millat University, Pitras Bukhari Road, H-8/4, Islamabad 44000, Pakistan; 6 Department of Vascular Surgery, Shifa International Hospital Pitras Bukhari Road, H-8/4, Islamabad 44000, Pakistan

## Abstract

Background: Lipoprotein(a) [Lp(a)] is a highly atherogenic particle identified as an independent risk factor for the development of atherosclerotic cardiovascular disease (ASCVD). This study aimed to investigate the frequency of Lp(a) testing and the incidence of elevated Lp(a) levels in the Pakistani population.

Methods: For this observational study, Lp(a) and lipid profile data from five years (June 2015 to October 2020) were acquired from the electronic patient records of a diagnostic laboratory with a countrywide network. The association of age and total cholesterol (TC), high-density lipoprotein cholesterol (HDL-C), low-density lipoprotein cholesterol (LDL-C), non-HDL, and triglyceride (TG) levels with two thresholds for Lp(a), that is, <30 mg/dL and ≥30 mg/dL, was calculated using the Kruskal-Wallis test, while the association between Lp(a) levels and lipid variables was calculated using Spearman correlation.

Results: For five years, 1060 tests were conducted, averaging 212 tests per year. Of these, 37.2% showed Lp(a) levels above 30 mg/dL. No significant differences were observed in the results between males and females. However, younger individuals displayed significantly higher Lp(a) levels. Additionally, there was only a weak correlation between the Lp(a) levels and other lipid variables.

Conclusion: Despite being recognized as a risk factor for ASCVD in the Pakistani population, only a small proportion of the large population underwent Lp(a) testing. Moreover, a significant proportion of the population exceeded this threshold.

## Introduction

Lipoprotein(a) [Lp(a)] is a macromolecular structure comprising a lipid core of cholesteryl esters and triacylglycerols surrounded by an outer shell of phospholipids, free cholesterol, and apolipoprotein B-100 (apoB-100) particles linked to apolipoprotein a [apo(a)] glycoprotein^1,2^. Lp(a) is synthesized exclusively in hepatocytes and is a major carrier of oxidized phospholipids (OxPLs) that can trigger multiple pro-inflammatory pathways^3,4^. Circulating levels of Lp(a) are determined by the *LPA* gene locus and are not influenced by dietary or environmental factors^5^. Data from randomized control trials have shown that diets lower in saturated fats, hormone replacement therapy (HRT), and liver disease result in lowered Lp(a) levels, whereas kidney disease results in a marked elevation of Lp(a)^6^.

Evidence from several studies suggests that elevated Lp(a) level is an independent risk factor for the development of ASCVD, including aortic valve stenosis, coronary heart disease, myocardial infarction, and stroke^7–12^. Several international guidelines have included Lp(a) testing in their recommendations, particularly for individuals with a high risk of cardiovascular diseases^13–19^. Plasma Lp(a) levels are genetically determined and generally remain stable; however, genetic variability exists among different ethnic groups, with greater levels observed in Africans than in Caucasians, Hispanics, and Asian populations^20,21^. Generally, an Lp(a) concentration of 50 mg/dL is considered a high-risk threshold^22^. High Lp(a) levels (>50 mg/dL) are estimated to be prevalent in 20% of the population worldwide^5^. Elevated Lp(a) levels have been identified as a causal risk factor in the Pakistani population; however, Lp(a) testing is not considered in routine ASCVD diagnosis^23,24^.

This study aimed to investigate the incidence of Lp(a) testing and elevated Lp(a) levels in a Pakistani population.

## Methods

### Study population

For this retrospective study, anonymized Lp(a) data from individuals referred for lipid testing between June 2015 and October 2020 were acquired from the electronic patient records of a diagnostic laboratory operating collection centers throughout Pakistan.

### Lp(a) and lipid profile analysis

Lp(a) levels were measured using an immunoturbidimetric assay (Alinity c Lp(a) kit, Abbott Laboratories, Illinois, USA). The lipid profile data for low-density lipoprotein cholesterol (LDL-C), high-density lipoprotein (HDL), total cholesterol (TC), and triglycerides (TG) were measured using a homogenous assay (Abbott Alinity CI analyzer).

### Statistical analysis

The data for study variables, including age, Lp(a), total cholesterol (TC), high-density lipoprotein (HDL), low-density lipoprotein (LDL), non-HDL, and triglyceride (TG) levels, were reported using descriptive statistics (minimum, maximum, median, and interquartile range [IQR]). The association between Lp(a) levels and sex was calculated using the Mann–Whitney U test. The level of significance was set at *p*¡0.05. Kruskal-Wallis tests were used to evaluate differences in Lp(a) levels among age groups and to analyze the association between age and lipid parameters (TC, HDL, LDL, non-HDL, and TG) with two Lp(a) thresholds (<30 mg/dL and ≥30 mg/dL). All statistical analyses and data visualizations were performed using SPSS version 27.

**Table 1:** Data variables of the study.

**Data characteristics**	**Minimum**	**Maximum**	**Median**	**IQR**
Age (*n* = 1060)	3.0	98.0	47.0	37.0–58.0
Lp(a) (*n* = 1060)	1.3	281.3	20.7	9.8–43.8
TC mg/dL (*n* = 190)	104.0	1010.0	192.0	156.7–222.5
HDL-C mg/dL (*n* = 190)	18.0	86.0	39.0	32.0–46.2
LDL-C mg/dL (*n* = 190)	50.0	800.0	123.0	94.7–162.2
Non-HDL mg/dL (*n* = 190)	62.0	990.0	149.5	114.7–184.2
TG mg/dL (*n* = 190)	38.0	891.0	134.5	97.0–215.0
Lp (a) (*n* = 190)^*^	1.3	281.3	19.2	9.2–47.4
Age (*n* = 190)^*^	3.0	83.0	41.5	30.7–52.0

**Notes.**

IQR Interquartile range Lp (a) Lipoprotein (a) TC Total cholesterol TG Triglycerides HDL-C High-density lipoprotein cholesterol

* Lp(a) levels and age data were gathered from 190 patients who had complete lipid profiles out of a larger group of 1060 patients with Lp(a) data.

**Figure 1.**
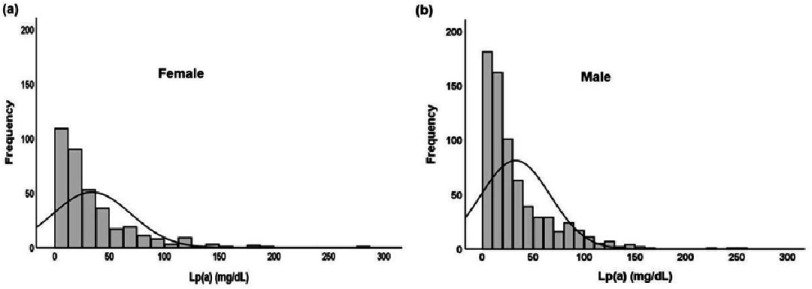
Gender-based Lp(a) frequency distribution in the Pakistani population, a) females, b) males.

## Results

In total, 1,060 individuals were included in this study. Lipid profiles were measured in 190 patients. Details of the study variables, such as age, Lp(a), TC, HDL, LDL, non-HDL, and TG, are presented in Table 1. The median age of participants was 47 years. The median Lp(a) level was 20.75 (9.8–43.85) mg/dL. Among these, 37.5% (*n* = 395) had Lp(a) levels > 30 mg/dL, while 21.3% (*n* = 225) had Lp(a) levels > 50 mg/dL. The data for lipid variables were available for 190 individuals, including the median levels of TC, HDL, LDL, and non-HDL. was obtained (Table 1).

**Figure 2.**
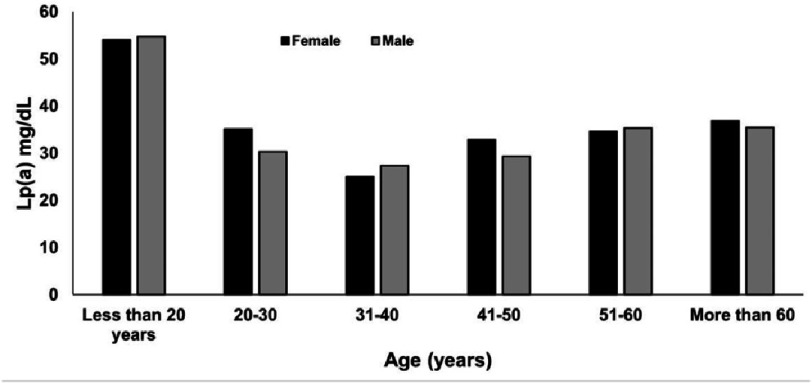
Trends of Lp(a) levels in different age groups of males and females.

**Figure 3.**
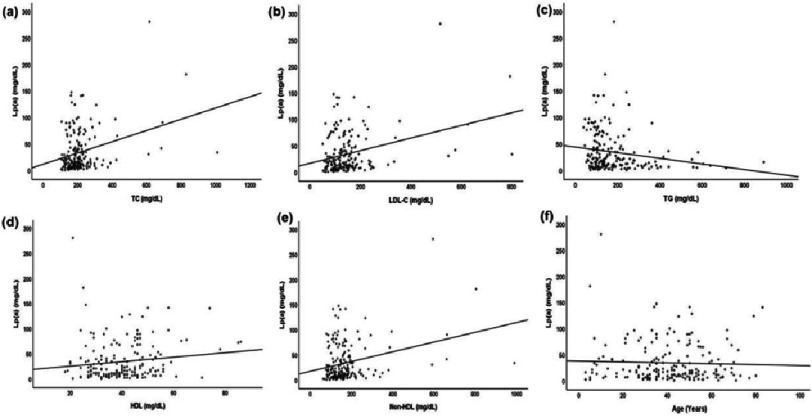
Spearman correlation between Lp(a), age, and lipid variables.

The levels of Lp(a) were not significantly different between sexes ( *p* = 0.45). Figure 1 shows the frequency distribution of Lp(a) levels in both male and female participants. The levels of Lp(a) were compared across various age groups, including those under 20 years, between 20 and 40 years, between 40 and 60 years, and over 60 years. The results showed significant differences between the groups ( *p* = 0.03) (Table 2). The median Lp(a) level for those ages below 20 years was 31.2 (9.1–92.2) mg/dL, while 23.3 (10.2–47.6) mg/dL for those aged above 60 years. Higher Lp(a) levels were observed in the younger population aged less than 20 years (Figure 2). Significant differences were observed for TC, LDL, and TG when comparing the association of the study variables among the thresholds of Lp(a). No association was found between age, HDL or non-HDL levels, and Lp(a) thresholds (Figure 3, Table 3).

**Table 2:** Association of gender and age with lipoprotein (a) levels.

**Data variables**		**N (%)**	**95% CI (Q1-Q3)**	**Lp(a) levels** **Median (IQR)**	***p* value**
Gender	Male	696.0 (76.0)	30.1–35.2	20.4 (9.6–43.3)	0.45
	Female	364.0 (34.0)	30.2–37.6	21.8 (9.9–44.7)	
Age (years)	<20	43.0 (4.0)	36.6–73.6	31.2 (9.1–92.2)	0.03
	20–40	287.0 (27.0)	25.8–33.3	18.7 (8.5–37.5)	
	40–60	501.0 (47.0)	29.1–34.7	20.9 (10.1–43.2)	
	>60	229.0 (22.0)	31.1–40.5	23.3 (10.2–47.6)	

**Notes.**

IQR Interquartile range CI Confidence Interval

## Discussion

**Figure 4.**
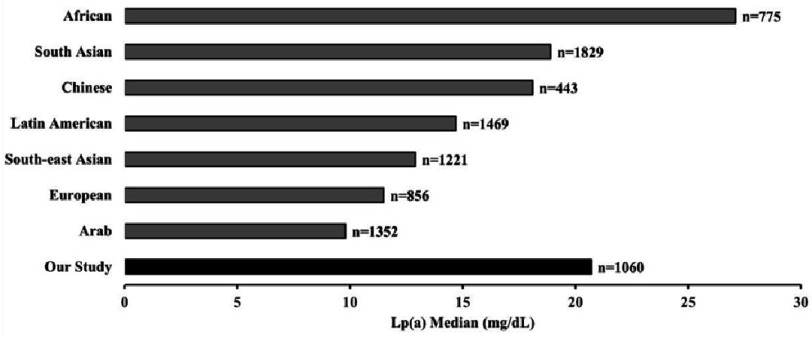
Median Lp(a) median values reported in the present study compared to the levels reported in other ethnicities.

This study presents the levels and incidence of Lp(a) testing in Pakistan. The results show that only 1060 tests were performed over five years. Among those tested, 21.2% had Lp(a) levels above 50 mg/dL, which is identified as a risk threshold based on the European Atherosclerosis Society/European Society of Cardiology (EAS/ESC) guidelines, while 37.2% had Lp(a) levels above 30 mg/dL, which is categorized as a risk category according to the American Heart Association (AHA) guidelines^14,16^. The median Lp(a) level of individuals included in the present study was 20.75 mg/dL. Previously, for Pakistani population higher mean levels were observed for those with acute coronary syndrome (47.03 mg/dL, *n* = 90) and diabetes (47.65 mg/dL, *n* = 68)^25,26^. Plasma Lp(a) concentration vary considerably among different racial and ethnic groups, where individuals of African ancestry have the highest levels while East Asians tend to have the lowest levels, while South Asians, Whites and Hispanics have intermediate levels^22,27,28^. These differences can be attributed to kringle IV polymorphisms in the *LPA* gene that code for apo(a), leading to wide variability in Lp(a) size within the population and among different ethnic groups^29^. In the present study, slightly higher levels were observed in the study population, compared to those observed in the Southeast Asian population (Figure 4)^30^. As the data were obtained from a referral laboratory, this might explain the slightly higher levels.

**Table 3:** Association of age, TC, HDL, LDL, non-HDL, and TG with Lp(a) thresholds i.e., <30 mg/dL and ≥30 mg/dL.

	**Lp(a)** **<30 mg/dL (*n* = 119)**		**Lp(a)** **≥30 mg/dL (*n* = 71)**		
***N* = 190**	**Median**	**IQR (Q1-Q3)**	**Median**	**IQR (Q1-Q3)**	**p value**
Age (Years)	42.0	31.0–52.0	40.0	30.0–51.0	0.75
TC (mg/dL)	183.0	150.0–216.0	204.0	172.0–239.0	0.01
HDL-C (mg/dL)	38.0	32.0–45.0	42.0	32.0–48.0	0.10
LDL-C (mg/dL)	114.0	93.0–152.0	135.0	103.0–174.0	0.01
Non-HDL (mg/dL)	147.0	112.0–181.0	159.0	124.0–194.0	0.07
TG (mg/dL)	142.0	102.0–242.0	115.0	92.0–179.0	0.02

**Notes.**

IQR Interquartile range TC Total cholesterol TG Triglycerides HDL-C High-density lipoprotein cholesterol LDL-C Low density lipoprotein cholesterol

The results of the present study show that Lp(a) levels were significantly higher in younger individuals than in those aged 60 years or above. Similar results were observed in a study conducted in a multi-ethnic population with a history of ASCVD^31^. Since elevated Lp(a) levels result in premature ASCVD, this could be the reason for higher Lp(a) observed in younger population. Moreover, the levels of Lp(a) were weakly correlated with other lipid variables such as TC, HDL, LDL-C, and TG. Previous studies have shown a weak correlation between Lp(a) levels and other lipid variables^32,33^.

However, higher Lp(a) levels were observed with higher LDL-C levels. A similar relationship was demonstrated in previous studies^34^. Triglyceride levels were negatively correlated with Lp(a) levels which is consistent with the results of other studies^32,35^. This could be either due to binding of apo(a) to the apoB of VLDL particles during the VLDL synthesis leading to the synthesis of VLDL-like particles that can be further metabolized by lipolysis or this could be due to Lp(a) metabolism by enzymes involved in TG and VLDL metabolism^36–38^.

Specific Lp(a) lowering therapies are not available yet and are still in clinical trials^39^. Statins remain the first line of lipid lowering therapy however, statins can significantly increase plasma Lp(a) levels^40,41^. Other drugs including proprotein convertase subtilase/kexin type 9 inhibitors (PCSK9i) and mipomersen have the potential to lower Lp(a) levels^42,43^. The emerging mRNA therapies have also shown their effectiveness to lower Lp(a) levels in clinical trials^44–46^. Due to the nature of the present study, the data on lipid lowering drugs is not available, therefore this aspect could not be addressed in the present study.

Several international guidelines recommend Lp(a) measurement at least once in the lifetime of an individual, however Lp(a) testing is not highly adopted worldwide and heterogeneity regarding the incorporation of Lp(a) testing in patient care still exists^47–50^. In Pakistan, where cardiovascular diseases remain the leading cause of deaths, early screening of risk factors in the population can help prevent cardiovascular events. Since, Lp(a) remains an independent causal factor for ASCVD, it is imperative that Lp(a) testing is conducted at a large scale. It is evident from the present study, only a fraction of population had their Lp(a) levels tested, therefore measures should be taken to ensure Lp(a) testing at least once preferably with the first lipid profile. This will not only aid physicians to stratify the risk of cardiovascular events but will also be helpful in reducing the burden of cardiovascular diseases.

There are some limitations of the present study. The major one is that since the data was obtained from a referral laboratory, details of the disease status such as presence of ASCVD, diabetes, kidney or liver disease of the individuals were not available, therefore the risk and impact of these conditions on Lp(a) levels cannot be ascertained*.* Moreover, the study presents a cohort referred for Lp(a) testing which could have introduced a selection bias limiting the generalizability of our findings. As mentioned earlier, due to the nature of the data utilized for this study, the details of the medications and their impact on Lp(a) levels could not be elucidated.

## Conclusion

The present study showed that only a small fraction of the population of Pakistan is registered for Lp(a) testing. A significant proportion of our study population had Lp(a) levels above the suggested threshold. Being a country with the highest rate of mortality due to cardiovascular diseases, it is imperative to screen the general population for ASCVD risk stratification, and the inclusion of mandatory Lp(a) testing can be helpful in early screening. There is a dire need for education regarding the recommended guidelines for Lp(a) testing and the appropriate clinical management of patients with elevated Lp(a) levels. Additionally, public health policies should be developed and implemented to address this issue, focusing on increasing awareness, improving screening practices, and ensuring access to appropriate interventions

## Authors’ contributions

**Conceptualization:** Fouzia Sadiq. **Methodology:** Hijab Batool, Omar R. Chughtai, and Muhammad D. Khan. **Resources:** Hijab Batool, Quratul Ain, Omar R. Chughtai, and Muhammad D. Khan. **Supervision:** Mohammad I. Khan and Fouzia Sadiq. **Validation:** Hijab Batool. **Visualization:** Madeeha Khan and Quratul Ain. **Writing - original draft:** Hijab Batool, Madeeha Khan, and Quratul Ain. **Writing - review & editing:** Hijab Batool, Madeeha Khan, Quratul Ain, Omar R. Chughtai, Muhammad D. Khan, Mohammad I. Khan, and Fouzia Sadiq.

## Acknowledgement

We are thankful to Mr. Amjad Nawaz, Shifa Tameer e Millat University, Islamabad, for helping in conducting statistical analysis.
